# Investigation of the role and mechanism of ARHGAP5-mediated colorectal cancer metastasis

**DOI:** 10.7150/thno.43427

**Published:** 2020-05-01

**Authors:** Tian Tian, Zhan-Hong Chen, Zongheng Zheng, Yubo Liu, Qi Zhao, Yuying Liu, Huijun Qiu, Qian Long, Miao Chen, Liren Li, Fangyun Xie, Guangyu Luo, Xiaojun Wu, Wuguo Deng

**Affiliations:** 1 Sun Yat-sen University Cancer Center, State Key Laboratory of Oncology in South China, Collaborative Innovation Center for Cancer Medicine, Guangzhou 510060, China; 2 The Third Affiliated Hospital, Sun Yat-sen University, Guangzhou, 510630, China

**Keywords:** colorectal cancer, tumor metastasis, ARHGAP5, CREB1, miR-137

## Abstract

**Background:** Metastatic colorectal cancer (CRC) is a lethal disease; however, the underlying molecular mechanisms remain unclear and require further study.

**Methods:** RNA-Seq, PCR, Western blotting, immunohistochemistry, ChIP and RNAi assays were performed to investigate Rho GTPase-activating protein 5 (ARHGAP5, aslo known as p190RhoGAP-B, p190-B) expression and the clinical relevance, functional roles and regulatory mechanisms of this protein using human CRC cells and tissues. *In vivo*, two cell-based xenograft models were used to evaluate the roles of ARHGAP5 in CRC metastasis.

**Results:** Here, we report that ARHGAP5 expression is significantly increased in metastatic CRC tissues and is inversely associated with patient overall survival. The suppression of ARHGAP5 reduces CRC cell metastasis *in vitro* and in cell-based xenograft models. Furthermore, we show that ARHGAP5 promotes CRC cell epithelial-mesenchymal transition by negatively regulating RhoA activity. Mechanistically, cAMP response element-binding protein (CREB1) transcriptionally upregulates ARHGAP5 expression, and decreased miR-137 further contributes to ARHGAP5 mRNA stability in CRC.

**Conclusions:** Overall, our study highlights the crucial function of ARHGAP5 in CRC metastasis, thus suggesting novel prognostic biomarkers and hypothetical therapeutic targets.

## Introduction

Metastatic colorectal cancer (CRC) is a lethal disease that caused almost 861,000 deaths in 2018 worldwide 1, 2. The liver is the most common organ for distant metastasis in CRC patients. Clinically, approximately 20-25% of CRC patients present with synchronous liver metastases at diagnosis, while approximately 50% of CRC patients develop metachronous liver metastases within 3 years of treatment 3-5. Multidisciplinary team therapy is currently the main treatment mode for liver metastasis in CRC patients 4, 6. However, the 5-year survival rate of these patients is only 20%, while the 5-year survival rate is 90% for early-stage CRC patients 1. Therefore, investigations of the molecular mechanisms underlying CRC metastasis may help to develop novel prognostic biomarkers and therapeutic strategies for CRC patients.

RhoA, a member of the Rho family of small GTPases, is involved in regulating cell shape and movement through cytoskeletal remodeling, thus influencing cell migration and invasion 7. Accordingly, the activity of this protein is tightly controlled by guanine nucleotide-exchange factors (GEFs) and GTPase-activating proteins (GAPs) that determine whether GTP or GDP is bound to this protein. RhoA is activated by GEFs, which catalyze the release of GDP and thus allow GTP to bind this protein 8, 9. RhoA, in turn, is inactivated by GAPs, which bind to Rho proteins and induce them to hydrolyze their bound GTP to GDP. The GAPs of many small GTPases contain a conserved Arg residue that is essential for catalyzing nucleotide hydrolysis 10. Rho GTPase-activating protein 5 (ARHGAP5, aslo known as p190RhoGAP-B, p190-B), a member of the RhoGAP family, negatively regulates RhoA activity 11-13. Although previous studies suggested that ARHGAP5 may play an oncogenic role in tumor progression, its biological function and regulatory mechanisms in CRC are poorly understood.

In the present study, we aimed to discover the key molecules involved in CRC liver metastasis. We examined the clinical relevance, function and underlying regulatory mechanisms of ARHGAP5 in CRC metastasis. Additionally, our study suggests novel prognostic biomarkers and hypothetical therapeutic targets for metastatic CRC patients.

## Materials and Methods

### Patients and cells

Archived CRC tissue specimens (n=423) were collected after obtaining written informed consent, in accordance with our Institutional Review Board and the Declaration of Helsinki (Table S1). CRC, immortalized colon epithelial and 293T cell lines were purchased from ATCC (Manassas, VA, USA) and cultured under the conditions specified by the supplier. The cell lines SW480 derived from primary tumor and SW620 from a metastatic site in the same patient 14. All cells were negative for mycoplasma contamination and were authenticated based on short tandem repeat DNA fingerprinting before use.

### Immunoblotting and immunohistochemical (IHC) analysis

Immunoblotting and IHC analysis were conducted with standard procedures, as previously described 15, 16. Blotting membranes were stripped and reprobed with anti-β-Actin antibody as a loading control. The degree of immunostaining of formalin-fixed, paraffin-embedded sections was reviewed and scored independently by two pathologists based on both the proportion of positively stained tumor cells and the intensity of the staining. The following antibodies were used for immunoblotting or IHC analysis: ARHGAP5 (#2562, WB, 1:1000), Vimentin (#5741; WB, 1:1000; IHC, 1:200), N-cadherin (#13116; WB, 1:1000; IHC, 1:100), E-cadherin (#14472; WB, 1:1000; IHC, 1:100), RhoA (#2171; WB, 1:1000), CREB1 (#9197; WB, 1:1000; IHC, 1:6000), β-Actin (#3700; WB, 1:3000) (all from Cell Signaling, Beverly, USA) and ARHGAP5 (#ab199160; IHC, 1:100) (Abcam, Cambridge, MA, USA).

### Migration and invasion assays

The effects of ARHGAP5 on the migration and invasion of CRC cells were tested using transwell chambers, as previously reported 17, 18. Briefly, CRC cells were harvested and suspended in 200 μL serum-free medium (2×10^5^) and were plated in the top chamber with (migration) or without (invasion) a Matrigel-coated membrane (pore size, 8 µm)( BD Biosciences, San Jose, USA). The lower chambers were filled with serum as a chemoattractant. The cells were incubated for 24 h, and cells that did not migrate through the pores were removed with a cotton swab. Invading cells were fixed, stained with crystal violet (Sigma-Aldrich, St. Louis, USA) and counted in five random fields.

### RhoA activity assay

Rho activity was assessed using an Active Rho Detection Kit (#8820, Cell Signaling, Beverly, USA). The measurement of GTPase activity was based on the ability of the GTP-bound (active) form to bind the Rhotekin-RBD fusion protein, which could then be immunoprecipitated with glutathione resin. Total RhoA levels were similarly analyzed using an aliquot of whole-cell lysate. Active RhoA and total RhoA were analyzed by immunoblotting analysis with an anti-RhoA antibody that was included in the kit.

### Animal study

All animal experiments were performed in accordance with a protocol approved by our institutional Animal Care and Use Committee. Female BALB/c nude mice (4/5 weeks old) were obtained from the Animal Center of Guangdong Province (Guangzhou, China). To evaluate the effect of ARHGAP5 on CRC metastasis* in vivo*, two xenograft models were used. For liver metastasis, the mice were anesthetized, and CRC cells (2 × 10^6^ per mouse) were injected into the distal tip of the spleen using a Hamilton syringe (8 mice/group). These mice were sacrificed six weeks postinjection. The spleen and liver were recovered, paraffin-embedded and stained with H&E. The micrometastases in the livers were examined and counted under a dissecting microscope. For mesenteric metastasis, mice were anesthetized, and CRC cells (2×10^6^ per mouse) were orthotopically implanted into the cecum (8 mice/group). The mice were sacrificed, the intestines were removed, and the metastatic nodules in the intestines were counted after 6 weeks.

### Statistical analysis

To identify the significant differences between two groups, a Student's *t*-test was used. Survival curves were plotted using the Kaplan-Meier method and compared by the log-rank test. For correlation analysis between two continuous variables, *r* values represent Pearson's correlation coefficients, and *p*-values were calculated by Pearson's correlation test. For the study of the association between the expressions of two genes, the Chi-square test was used. Statistical analyses were performed with GraphPad version 5.0. A *P* value of less than 0.05 was statistically significant. All statistical tests were two-sided.

The details of RNA extraction and qPCR analysis, lentiviral transduction, immunofluorescence analysis, chromatin immunoprecipitation (ChIP) and luciferase promoter assays are described in the Supplementary Materials and Methods.

## Results

### Increased ARHGAP5 expression is associated with CRC metastasis and poor prognosis

**Figure 1:**
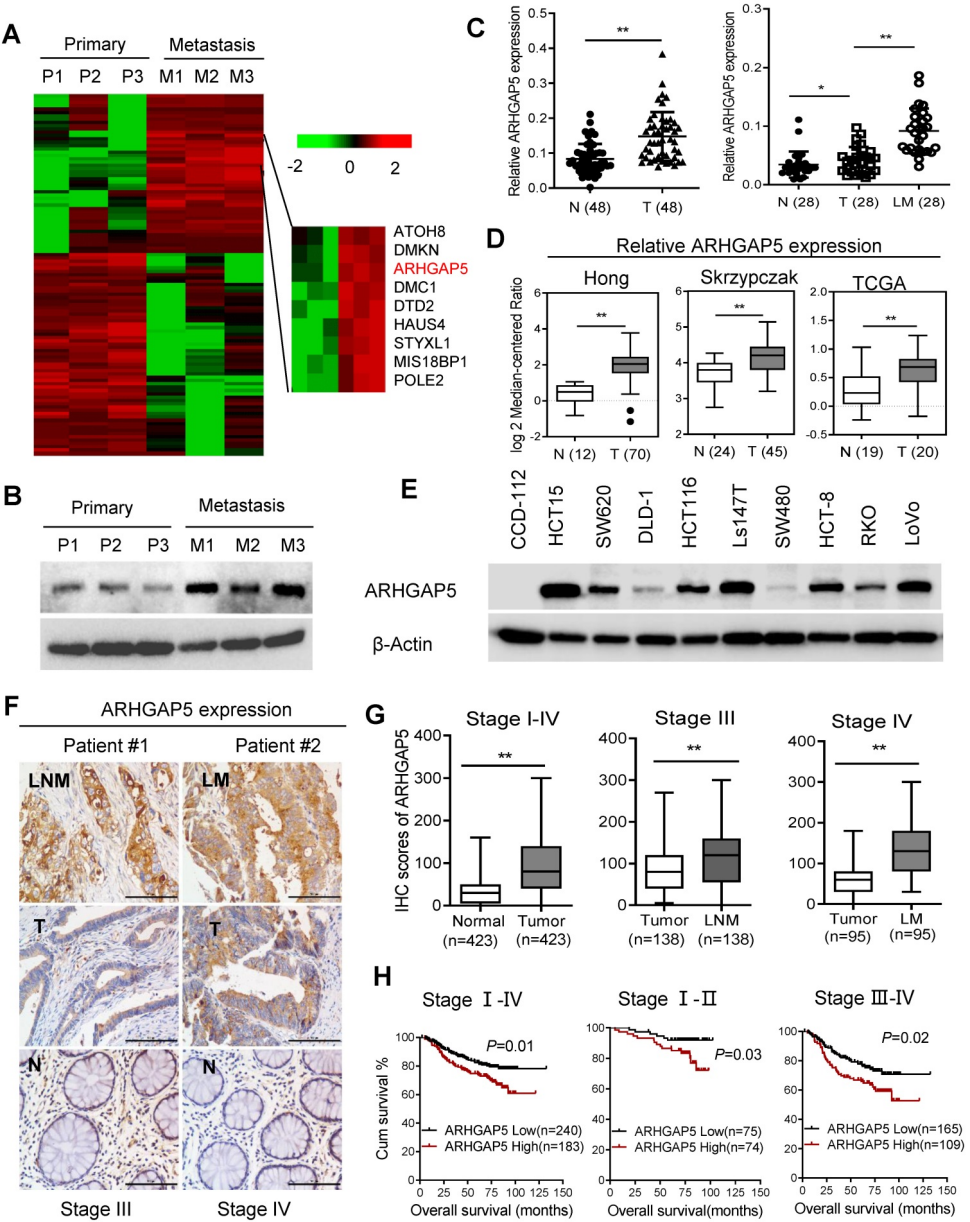
** Increased ARHGAP5 expression is associated with CRC metastasis and poor prognosis. (A)** Heatmap profiling the gene expression of paired primary and liver metastasis CRC tissues (n=3), as analyzed by RNA-seq. **(B)** Immunoblotting analysis of ARHGAP5 expression in paired primary and liver metastasis CRC tissues. β-Actin was included as a loading control. **(C)** qPCR analysis of ARHGAP5 expression in 48 pairs of CRC tumor (T) and adjacent normal issues (N) and in 28 pairs of liver metastases (LM) and primary (T) tissues. **(D)** ARHGAP5 expression in multiple CRC microarray data sets available from the Oncomine database (www.oncomine.org). **(E)** Immunoblotting analysis of ARHGAP5 expression in CRC cells and epithelial colorectal cells (CCD112). **(F-G)** Representative IHC staining and quantification of ARHGAP5 in paired primary CRC tumor (n=423), lymph node metastatic (LNM, n=138) or liver metastatic tissues (LM, n=95). **(H)** Kaplan-Meier analysis of the overall survival (OS) or disease-free survival (DFS) of CRC patients based on ARHGAP5 expression (log-rank test). Data in C, D, and G are presented as the mean ± the SD. *P < 0.05, **P < 0.01 (Student's *t*-test).

To explore the key molecules that modulate CRC hepatic metastasis, we performed RNA sequencing (RNA-Seq) analysis of three paired primary and liver metastasis CRC tissues. RNA-Seq analysis and validation with immunoblotting showed that ARHGAP5 was markedly overexpressed in liver metastatic tissues compared to matched primary tumor tissues (Figure 1A, 1B). ARHGAP5 and ARHGAP35 (p190RhoGAP-B, P190A) are known as the main negative regulator of RhoA 19, and ARHGAP35 degradation is implicated in metastatic CRC 20. However, ARHGAP35 expression was not changed significantly in our RNA-seq analyses. PCR analysis also confirmed that ARHGAP5 was significantly upregulated in CRC liver metastatic tissues and primary tumor tissues compared to matched adjacent-normal tissues (Figure 1C). Furthermore, the overexpression of ARHGAP5 in CRC was also supported by the Oncomine database, including the Hong, Skrzypczak and TCGA datasets (Figure 1D). qPCR and immunoblotting analysis showed that the ARHGAP5 mRNA and protein levels were notably increased in CRC cells compared with colorectal epithelial cells (Figures 1E, S1). Consistently, the IHC analysis of tissue microarrays found that the ARHGAP5 expression levels were significantly upregulated in liver and lymph node metastatic tissues compared with paired primary tissues (Figure 1F-G). Strikingly, Kaplan-Meier survival analysis indicated that patients with high ARHGAP5 expression levels had a shorter overall survival (OS) and disease-free survival (DFS) (Figure 1H). Multivariate analysis also indicated that ARHGAP5 expression was an independent prognostic factor in CRC patients (Table S2). These results indicate that ARHGAP5 may serve as a potential prognostic biomarker and may contribute to CRC metastasis.

### ARHGAP5 inhibition suppresses CRC metastasis *in vitro* and* in vivo*

**Figure 2:**
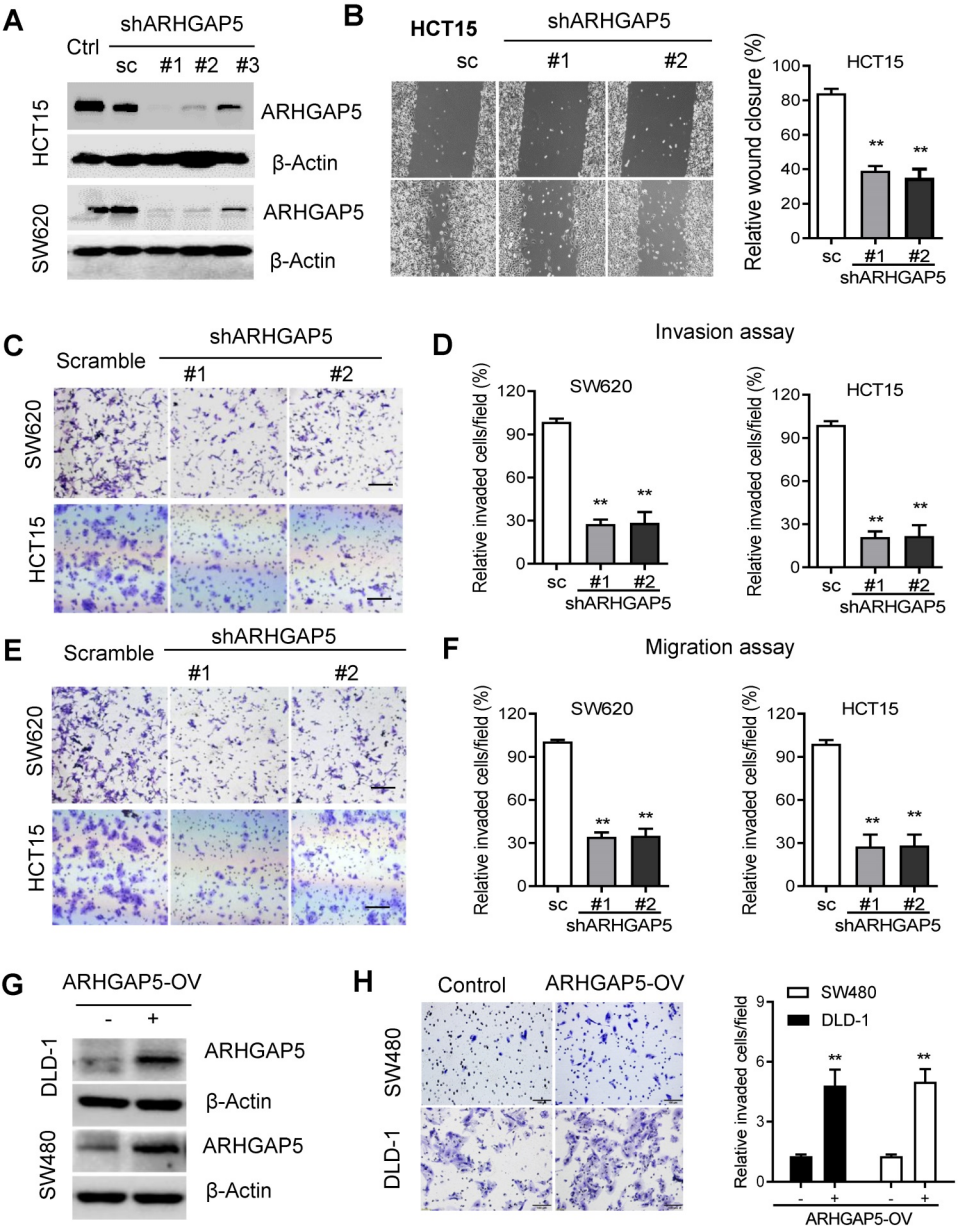
** ARHGAP5 inhibition suppresses CRC cell migration and invasion activity. (A)** Immunoblotting evaluating the knockdown efficiency of ARHGAP5 with three unique shRNAs (#1, #2 and #3) in SW620 and HCT15 cells. Scrambled shRNA (sc) was used as a negative control, and β-Actin was included as a loading control. **(B)** Representative images and quantification showing the wound healing of HCT15 cells with or without ARHGAP5 knockdown. **(C-F)** Representative images and quantification showing the cell invasion and migration of the indicated CRC cells with or without ARHGAP5 knockdown. **(G)** Immunoblotting analysis of ARHGAP5 expression in DLD-1 and SW480 cells overexpressing ARHGAP5 (ARHGAP5-OV). **(H)** Representative images and quantification showing the cell invasion of the indicated CRC cells overexpressing ARHGAP5. Data in B, D, F and H are presented as the mean ± the SD (n=3). **P < 0.01 (Student's *t*-test).

Given the prognostic value of ARHGAP5 and its correlation with metastasis in CRC, we next sought to determine whether ARHGAP5 contributes to metastasis by affecting cancer cell migration and/or proliferation, which are 2 critical determinants of metastasis. Then, we stably knocked down ARHGAP5 by the lentiviral infection of shRNA targeting ARHGAP5 in SW620 and HCT15 cells which expressed higher level of ARHGAP5 (Figures 1E, 2A, S2B). However, MTS assays and colony formation assays showed that ARHGAP5 knockdown had a small effect on CRC cell proliferation (Figure S1B). Interestingly, ARHGAP5 knockdown significantly inhibited CRC cell wound healing, migration and invasion (Figure 2B-F). On the other hand, the overexpression of ARHGAP5 significantly enhanced cell invasion in the DLD1 cell line and in SW480 cells which expressed lower level of ARHGAP5 (Figure 1E, 2G-H). These findings indicate that ARHGAP5 plays an important role in the enhancement of CRC metastatic ability *in vitro*.

**Figure 3:**
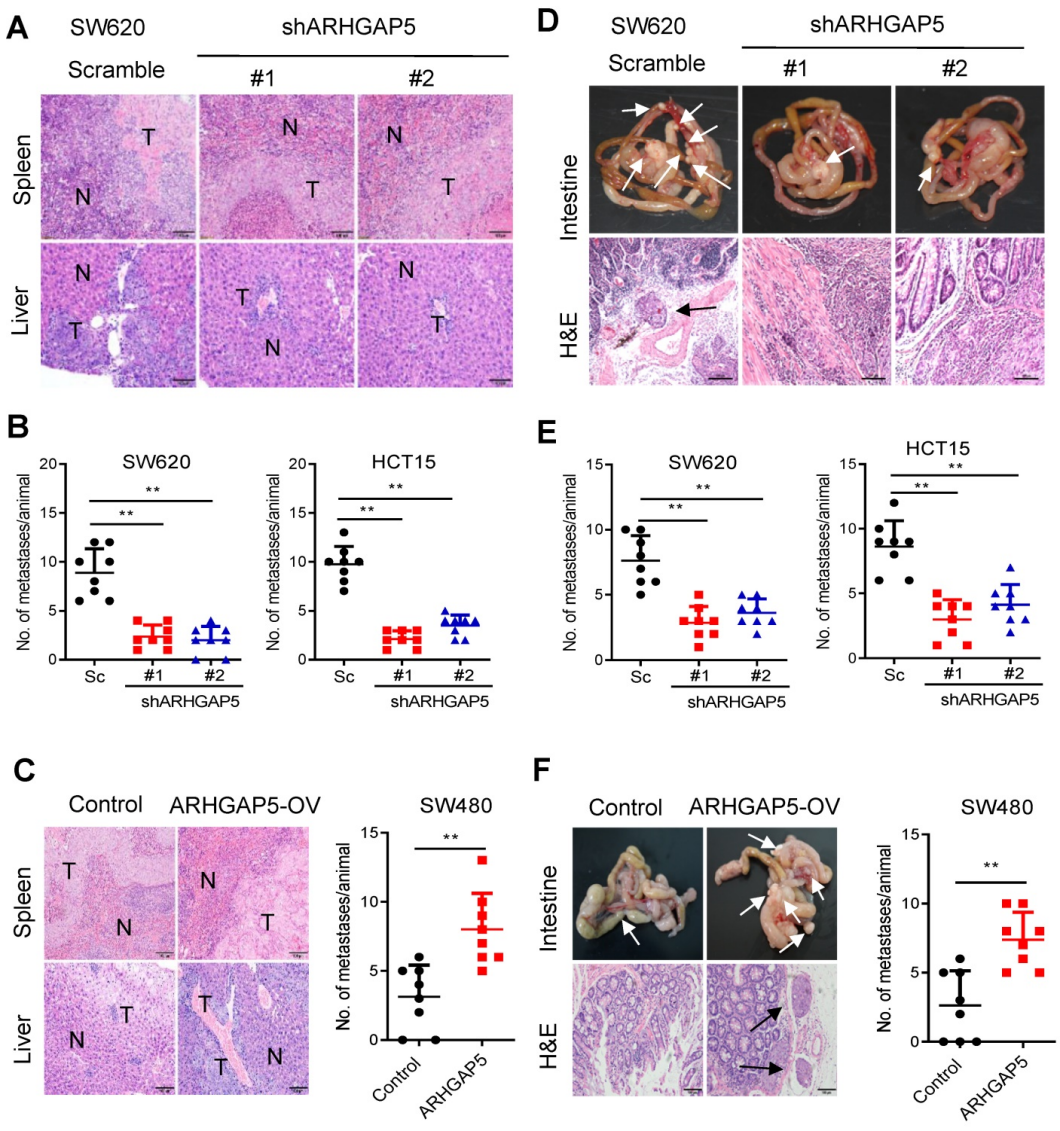
** Effects of ARHGAP5 knockdown or ARHGAP5 overexpression on CRC metastasis *in vivo*. (A-C)** Representative H&E staining and statistical results of the micrometastatic nodules in the liver from mice injected with the indicated cells into the spleen for 45 days. Scale bar: 100 µm, N=8 per group. **(D-F)** Representative H&E staining and statistical results of metastatic tumors in the excised intestines of mice orthotopically implanted with the indicated cells for 60 days. Scale bar: 100 µm, N=8 per group. White arrows indicate the metastatic foci. Data in B, C, E and F are presented as the mean ± the SD. **P < 0.01 (Student's *t*-test).

To further test whether ARHGAP5 contributes to CRC tumorigenesis *in vivo*, we performed cell-based xenograft experiments. For liver metastasis assays, the mice injected with control SW620 or HCT15 cells into the spleen had a heavy liver metastatic burden that was verified by histologic examination (H&E), whereas the knockdown of ARHGAP5 almost abolished this phenotype (Figure 3A, 3B). To further determine the effects of ARHGAP5 on the promotion of CRC metastasis, CRC cells were orthotopically implanted into the cecum of nude mice. The results indicated that ARHGAP5 suppression also reduced mesenteric metastatic nodules on the intestinal wall (Figure 3D, 3E). On the contrary, overexpressing ARHGAP5 in SW480 significantly enhanced the ability of these cells to form liver and mesenteric metastasis *in vivo* (Figure 3C, 3F). Overall, these results highlight the crucial roles of ARHGAP5 in promoting CRC metastasis* in vivo*.

### ARHGAP5 promotes EMT by negatively regulating RhoA activity

**Figure 4:**
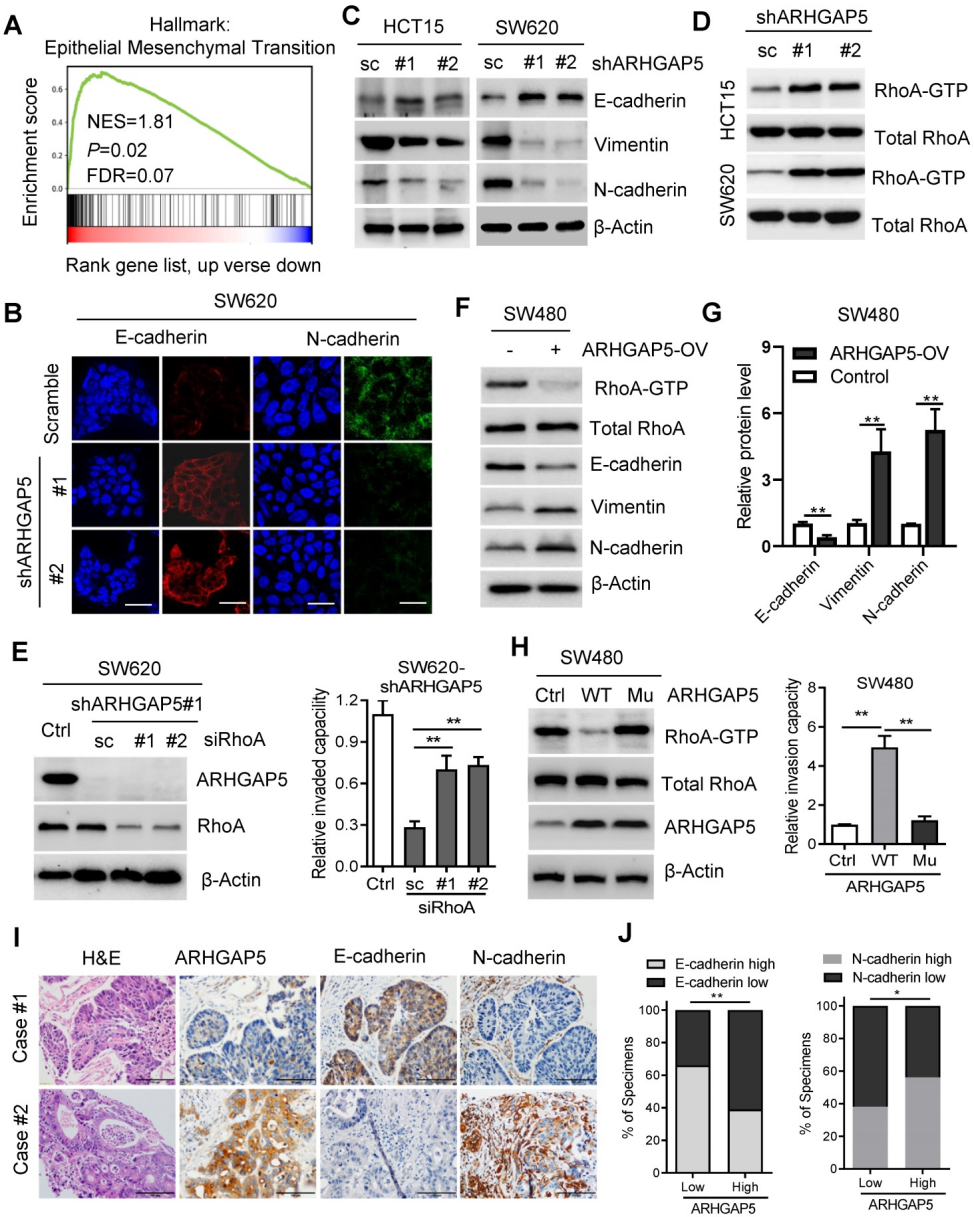
** ARHGAP5 negatively regulates RhoA activity. (A)** Gene set enrichment analysis (GSEA) demonstrating the enrichment of EMT-related gene sets in the ranked gene list of ARHGAP5 up versus ARHGAP5 down available from the TCGA CRC database.** (B)** Representative images of immunofluorescence staining for E-cadherin and N-cadherin expression in SW620 cells with ARHGAP5 knockdown (scale bar: 10 µm). **(C)** Immunoblotting analysis of E-cadherin, Vimentin and N-cadherin expression in the indicated CRC cells with ARHGAP5 knockdown. **(D)** Immunoblotting analysis of active RhoA (RhoA-GTP) pulled down by GS-RBD and total RhoA from whole-cell lysates in indicated cells with ARHGAP5 knockdown. **(E)** Immunoblotting analysis of ARHGAP5 and RhoA expression in SW620 cells with ARHGAP5 and/or RhoA knockdown, and quantification of the cell invasion capability of these cells. **(F)** Immunoblotting analysis of active RhoA (RhoA-GTP) pulled down by GS-RBD, total RhoA, E-cadherin, Vimentin and N-cadherin expression in SW480 cells with ARHGAP5 overexpression. **(G)** The expression levels of E-cadherin, Vimentin and N-cadherin were quantified and normalized to the corresponding levels of β-Actin as a loading control. **(H)** Immunoblotting analysis of active RhoA, total RhoA and ARHGAP5 expression in SW480 cells with wide type (WT) or mutant (Mu, R1297A) ARHGAP5 overexpression, and quantification of the cell invasion capability of these cells. **(I-J)** Representative images and the percentage of samples showing low or high ARHGAP5 expression relative to E-cadherin and N-cadherin. Scale bar: 100 µm, *P < 0.05, **P < 0.01 (Chi-square test).

Therefore, we further investigated the underlying mechanism of ARHGAP5 overexpression in CRC metastasis. Gene set enrichment analysis (GSEA) revealed that ARHGAP5 expression was positively corrected with the epithelial-mesenchymal transition (EMT) pathway in CRC (Figure 4A). Immunofluorescence analysis showed that the knockdown of ARHGAP5 significantly increased E-cadherin expression and decreased N-Cadherin expression in SW620 cells (Figure 4B), which was further verified by immunoblotting analysis (Figure 4C). As ARHGAP5 is a member of the RhoGAP family that negatively regulates RhoA activity 11, 12, we then detected total RhoA and active RhoA in SW620 and HCT15 cells with ARHGAP5 knockdown. The results indicated that the knockdown of ARHGAP5 obviously increased the amount of active RhoA (RhoA-GTP) pulled down by GS-RBD (Figure 4D). Furthermore, we found that knockdown of RhoA significantly restored CRC invasion capability that decreased due to ARHGAP5 depletion (Figure 4E). As expected, the overexpression of ARHGAP5 decreased active RhoA (RhoA-GTP) and E-cadherin expression but increased N-Cadherin and Vimentin expression compared with vector control SW480 cells (Figure 4F-G). These results also consistent with previous study that exogenous expression of constitutively active RhoA (RhoAQ63L) inhibited CRC and breast cancer cell invasion 21, 22. Also, we constructed a mutant ARHGAP5 (Mu) with a point mutation in conserved Arg residue (R1297A, Arg to Ala) referred to previous studies 23, 24. As shown, overexpression of mutant ARHGAP5 (Mu, R1297A) did not change RhoA activity and SW480 cell metastasis capability compared with vector control cells, further supporting that ARHGAP5 promotes CRC metastasis by suppressing RhoA activity (Figure 4H). Additionally, correlation studies in 423 CRC tissue specimens showed that ARHGAP5 expression was positively correlated with the expression of N-cadherin but was negatively correlated with the expression of E-cadherin (Figure 4I-J). Taken together, these results indicate that ARHGAP5 promotes EMT by negatively regulating RhoA activity in CRC.

### CREB1 transcriptionally upregulates ARHGAP5 expression in CRC

**Figure 5:**
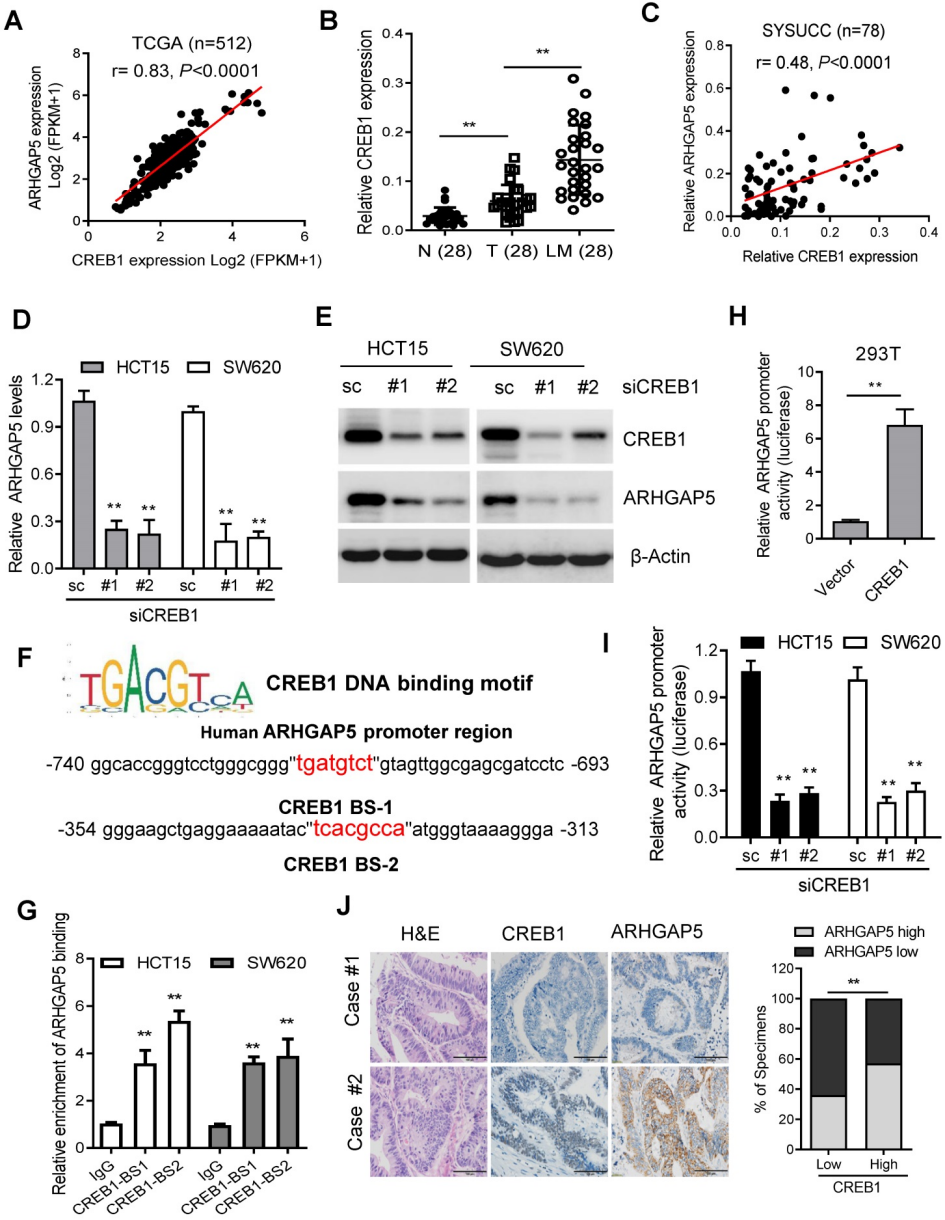
** CREB1 transcriptionally upregulates ARHGAP5 expression. (A)** Scatterplots of ARHGAP5 vs CREB1 mRNA expression in CRC samples available from the TCGA database (n=512). The Pearson correlation coefficient (r) and *P* value are displayed. **(B)** qPCR analysis of CREB1 mRNA levels in 28 pairs of CRC primary (T), liver metastasis (LM) and adjacent normal tissues (N). **(C)** Scatterplots of ARHGAP5 vs CREB1 mRNA expression in CRC samples available from SYSUCC (n=78). The Pearson correlation coefficient (r) and *P* value are displayed. **(D-E)** qPCR and immunoblotting analysis of ARHGAP5 expression in SW620 and HCT15 cells transfected with CREB1 siRNAs (#1 and #2). **(F)** Two CREB1 DNA-binding sites are present in the human ARHGAP5 promoter region. **(G)** ChIP-PCR analysis of CREB1 binding to the promoter region of ARHGAP5 in HCT15 and SW620 cells. **(H-I)** Relative ARHGAP5 luciferase promoter activity in the indicated cells with CREB1 overexpression or depletion. **(J)** Representative images and the percentage of samples showing low or high ARHGAP5 expression relative to CREB1. Scale bar: 100 µm, **P < 0.01 (Chi-square test). Data in B, D, G and H are presented as the mean ± the SD (n=3). **P < 0.01 (Student's *t*-test).

To assess the molecular regulation of ARHGAP5, we first surveyed genetic alterations of this gene using the cBioPortal datasets and found that the ARHGAP5 locus is unamplified in CRC, indicating that ARHGAP5 may be transcriptionally regulated (Figure S2A). Bioinformatics analysis with the JASPAR and TCGA databases predicted that cAMP responsive element binding protein (CREB1) was a potential transcription factor of ARHGAP5, and there was a significant, positive correlation between CREB1 mRNA and ARHGAP5 mRNA expression (Figure 5A). qPCR analysis revealed that CREB1 expression was significantly upregulated in CRC liver metastatic tissues and primary tumor tissues compared to matched adjacent normal tissues (Figure 5B), and the expression of CREB1 was tightly correlated with the expression of ARHGAP5 in CRC samples available from SYSUCC (n=78) (Figure 5C). In addition, qPCR and immunoblotting analysis indicated that the depletion of CREB1 obviously decreased the expression of ARHGAP5 in HCT15 and SW620 cells (Figure 5D-E). As CREB1 activates target genes through cAMP (Adenosine 3',5'-cyclic monophosphate) response elements 25, we also treated SW480 and DLD-1 cells with addition of exogenous cAMP. The results shows that ARHGAP5 expression was significantly increased by adding exogenous cAMP (Figure S2B-C). Through bioinformatics analysis, we also identified two CREB1 DNA-binding sites in the human ARHGAP5 promoter region (Figure 5F). ChIP-PCR assays showed that CREB1 can bind to the promoter region of ARHGAP5 in HCT15 and SW620 cells (Figure 5G). A dual-luciferase reporter assay showed that the relative ARHGAP5 luciferase promoter activity increased or decreased with CREB1 overexpression or depletion, respectively, in the indicated cells (Figure 5H-I). Additionally, correlation studies in 423 CRC tissue specimens showed that ARHGAP5 expression was positively correlated with the expression levels of CREB1 (Figure 5J). In summary, these results indicate that CREB1 transcriptionally upregulates ARHGAP5 expression in CRC.

### Decreased miR-137 contributes to ARHGAP5 overexpression in CRC

**Figure 6:**
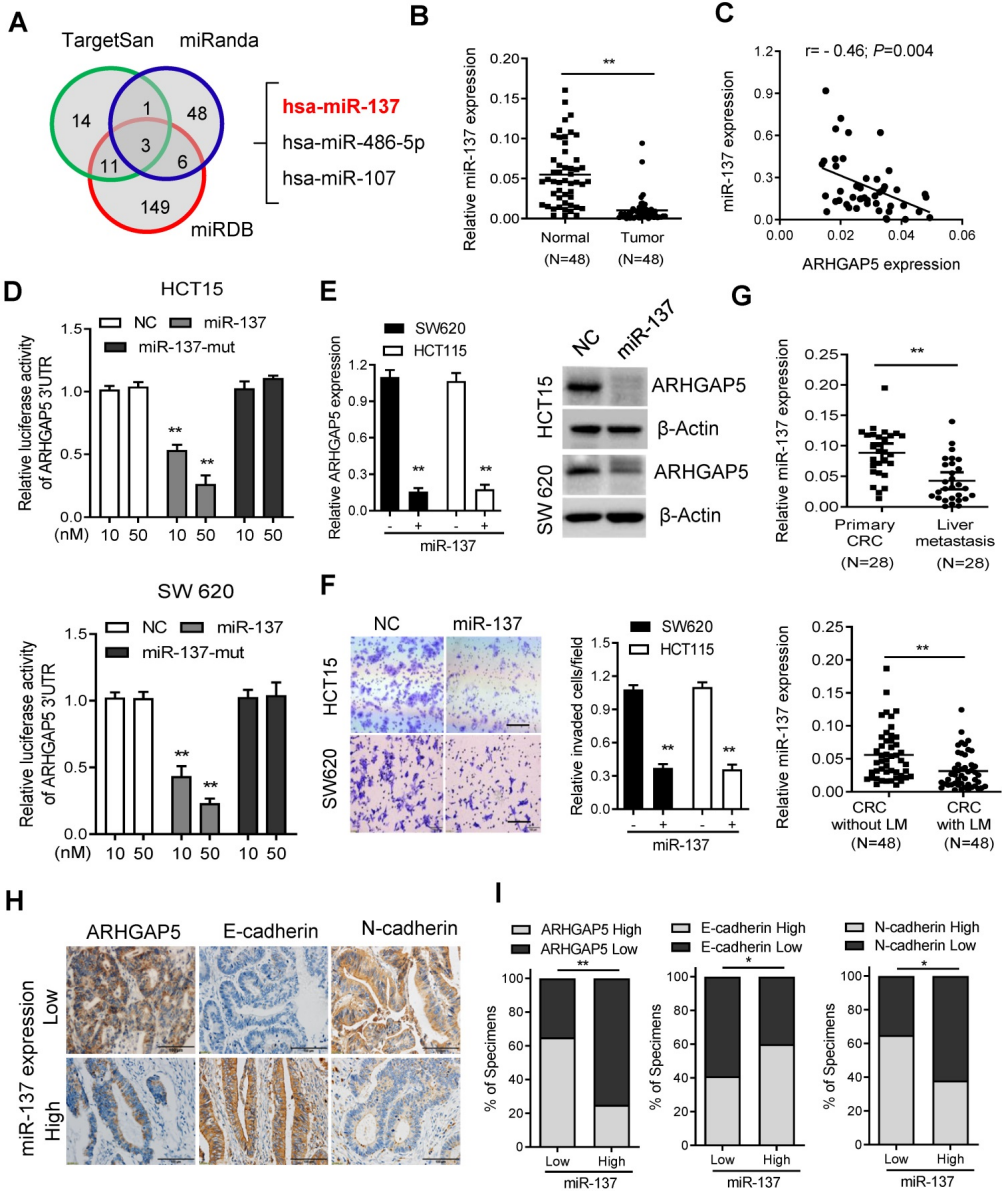
** Decreased miR-137 contributes to ARHGAP5 overexpression. (A)** Venn diagrams showing the number of potential miRNAs targeting the 3'UTR of ARHGAP5, as predicted by three databases. **(B)** qPCR analysis of miR-137 expression in paired CRC samples (n=48). **(C)** Scatterplots of ARHGAP5 vs miR-137 expression in CRC samples (n=48). The Pearson correlation coefficient (r) and *P* value are displayed. **(D)** Luciferase activity results of reporters containing the end of the 3'UTR of ARHGAP5 in cells transfected with a miR-137 mimic, miR-137 mutation mimics (miR-137-mut) and negative controls (NCs). **(E)** qPCR and immunoblotting analysis of ARHGAP5 expression in the indicated cells transfected with a miR-137 mimic. **(F)** Representative images and quantification of the effects of miR-137 on CRC cell invasion. Scale bar: 100 µm. **(G)** qPCR analysis of miR137 expression in 28 pairs of liver metastasis tissues and primary CRC tissues and in 48 pairs of CRC tissues with or without liver metastasis. **(H-I)** Representative images and the percentage of samples showing low or high miR137 expression relative to ARHGAP5, E-cadherin and N-cadherin. Scale bar: 100 µm, *P < 0.05, **P < 0.01 (Chi-square test). Data in D, E and F are presented as the mean ± SD (n=3). **P < 0.01 (Student's *t*-test).

As microRNAs (miRNAs) are small, noncoding RNAs that act as the master regulators of gene expression in CRC cells 26, 27, we then investigated whether ARHGAP5 was regulated by specific miRNAs. Analysis using publicly available algorithms (TargetScan, miRanda and miRDB) showed that ARHGAP5 was the predicted target of miR-137, miR-486-5P and miR-107 (Figure 6A). Although qPCR analysis showed that these three miRNAs were significantly downregulated in CRC tumor tissues, only miR-137 was significantly negatively correlated with ARHGAP5 mRNA expression (Figures 6B-C, S3A-B). Further analysis using the TargetScan algorithm showed that the 3'UTR (from 1041 to 1048 bp) of ARHGAP5 was a predicted target of miR-137 (Figure S3C). Moreover, a luciferase reporter assay showed that the overexpression of miR-137 repressed the luciferase activity of ARHGAP5-3'UTR in HCT15 and SW620 cells with low miR-137 expression (Figure 6D, S3D). However, ectopically expressing miR-137 mutation mimics did not inhibit the ARHGAP5-3'UTR luciferase activity (Figure 6D). Accordingly, the ectopic expression of miR-137 significantly decreased both ARHGAP5 expression (Figure 6E) and inhibited cell invasion (Figure 6F) in HCT15 and SW620 cells. Clinically, qPCR analysis showed that the miR137 level was significantly lower in CRC liver metastatic tissues than in paired primary tumor tissues and was significantly lower in CRC patients with liver metastasis than in CRC patients without liver metastasis (Figure 6G). IHC staining and statistical analyses further revealed that miR-137 expression was positively correlated with the expression of N-cadherin but was negatively correlated with the expression of ARHGAP5 and E-cadherin (Figure 6H-I). These results clearly demonstrate that decreased miR-137 contributes to ARHGAP5 overexpression and enhances CRC metastasis.

## Discussion

The distant metastasis of CRC patients is one of the most difficult challenges faced by clinicians. Progress has been made in the treatment of metastatic CRC in the past several decades due to the development of targeted drugs and immunotherapy 28. Combining chemotherapeutic cytotoxic drugs with biologic monoclonal antibodies (cetuximab and bevacizumab) provides clinical benefits for metastatic CRC patients 4, 29. The FDA recently approved immune checkpoint inhibitors (pembrolizumab and nivolumab) for the treatment of metastatic CRC patients with high microsatellite instability (MSI-H) 30, 31. However, the prognosis of metastatic CRC patients is far from satisfactory. In particular, there was a lack of efficacy of these antibodies in the majority of CRC patients with KRAS mutant-type or microsatellites stable (MSS) disease 32. Therefore, it is necessary to determine the underlying mechanisms, identify novel molecular biomarkers and develop appropriate therapies for metastatic CRC.

Epithelial to mesenchymal transition (EMT) is a developmental process of cell remodeling and is critical for embryogenesis, cell invasion and tumor metastasis 33, 34. Cancer-associated EMT is driven by key transcription factors that are finely regulated by multiple signaling cues and cofactors 35. In addition, the members of the Rho family of small GTPases, including RhoA, Rac1, Cdc42, etc., act as molecular switches that regulate cell shape, establish cell-cell junctional complexes and regulate the EMT process 36. ARHGAP35 and ARHGAP5 are two main GAPs regulating the Rho family of small GTPases 19. Previous studies have shown conflicting results that ARHGAP35 may be involved in CRC progression as an oncogene or tumor suppressor 20, 37, 38. ARHGAP5 has been identified as an oncogene in lung cancer, hepatocellular carcinoma (HCC) and gastric cancer 39-41, and a tumor suppressor gene in invasive epithelial ovarian cancer 42. In the current study, our results showed that ARHGAP5 also plays an important oncogenic role in the process of promoting CRC metastasis by both negatively regulating RhoA activity and promoting EMT. Although previous studies suggested that ARHGAP5 was associated with tumors, its underlying regulatory mechanisms in cancer cells are controversial and poorly understood. A previous study showed that ARHGAP5 was upregulated by the amplification of the chromosomal region 14q12 in a subgroup of hepatocellular carcinoma 40, by downregulation of miR-486-5p or miR-774 in lung cancer and nasopharyngeal carcinoma respectively 39, 43. We report here that ARHGAP5 is transcriptionally regulated by CREB1 and is posttranscriptionally controlled by miR-137 in CRC. Although both CREB1 and miR-137 have been reported to regulate CRC aggression and tumorigenicity 44, 45, our findings show that these molecules also synergistically promote CRC metastasis by regulating ARHGAP5.

The substantial body of evidence that shows that Rho GTPases are related to cancer has made the key components of Rho GTPase signaling attractive therapeutic targets for cancer drug discovery 46. A current promising approach is the development of small molecule inhibitors targeting protein kinase effectors upstream or downstream of Rho GTPase, and several inhibitors are currently in clinical trials for cancer treatment 47, 48. These targeted inhibitors may offer efficacious treatment options for future precision cancer therapy, particularly in combination with chemotherapy or immunotherapy agents 48. One of the remaining challenges is to better understand the detailed function and underlying regulatory mechanism of Rho GTPase signaling in the context of specific cancer types. Thus, this current study may not only provide a new understanding of CRC metastasis but also may enable the development of effective therapeutics for the treatment of metastatic CRC.

In conclusion, our findings reveal that ARHGAP5 is transcriptionally regulated by CREB1 and is post- transcriptionally controlled by miR- 137, and ARHGAP5 promotes CRC metastasis by negatively regulating RhoA activity. Additionally, our study suggests that ARHGAP5 might be used as a novel biomarker and therapeutic target for metastatic CRC patients.

## Supplementary Material

Supplementary figures and tables.

## Figures and Tables

**Figure 7:**
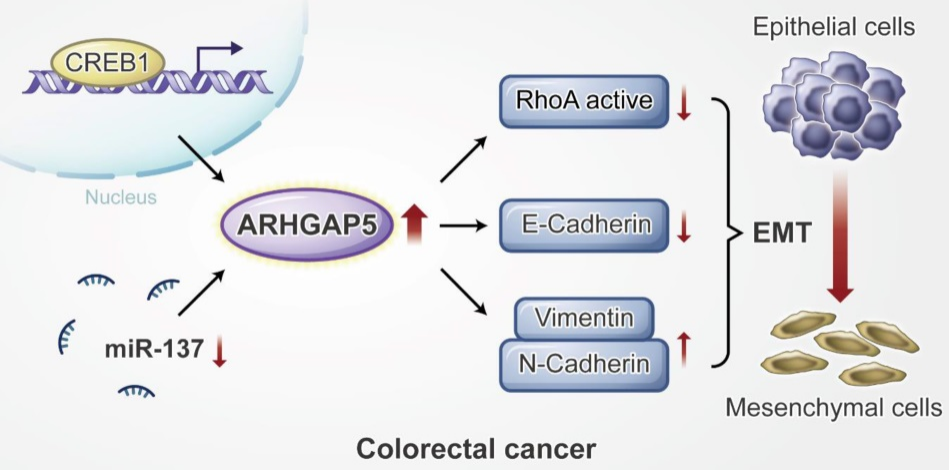
** Proposed working model of this study.** ARHGAP5 promotes CRC cell epithelial-mesenchymal transition by negatively regulating RhoA activity. ARHGAP5 is transcriptionally regulated by CREB1 and post-transcriptionally controlled by miR-137.

## Abbreviations

ARHGAP5: Rho GTPase-activating protein 5
CREB1: cAMP response element-binding protein
miR-137: mircro-RNA-137
CRC: colorectal cancer
GEF: guanine nucleotide-exchange factor
GAP: GTPase-activating protein
shRNAs: short hairpin RNAs
qPCR: quantitative real-time PCR
OS: overall survival
FISH: fluorescence *in situ* hybridization
ChIP: chromatin immunoprecipitation
RNAi: RNA interference
siRNAs: small interfering RNAs
H&E: hematoxylin and eosin
IHC: immunohistochemical
GSEA: Gene set enrichment analysis
RNA-seq: RNA sequencing
TCGA: The Cancer Genome Atlas
WT: wild-type
EMT: epithelial-mesenchymal transition
