# Investigation of the role and mechanism of ARHGAP5-mediated colorectal cancer metastasis: Erratum

**DOI:** 10.7150/thno.108943

**Published:** 2025-01-09

**Authors:** Tian Tian, Zhan-Hong Chen, Zongheng Zheng, Yubo Liu, Qi Zhao, Yuying Liu, Huijun Qiu, Qian Long, Miao Chen, Liren Li, Fangyun Xie, Guangyu Luo, Xiaojun Wu, Wuguo Deng

**Affiliations:** 1 Sun Yat-sen University Cancer Center, State Key Laboratory of Oncology in South China, Collaborative Innovation Center for Cancer Medicine, Guangzhou 510060, China; 2 The Third Affiliated Hospital, Sun Yat-sen University, Guangzhou, 510630, China

The authors regret that incorrect images appear in Figure 3F and 6F during data arranging processes. The reprehensive invasion image for NC group in Figure 3F, and reprehensive H&E staining for ARHGAP5-OV group in Figure 6F were incorrectly displayed. Now the corrected Figure 3F and 6F are shown below. The correction made in this erratum does not affect the original conclusions or any part of the text and figure legends.

## Figures and Tables

**Figure 3F:**
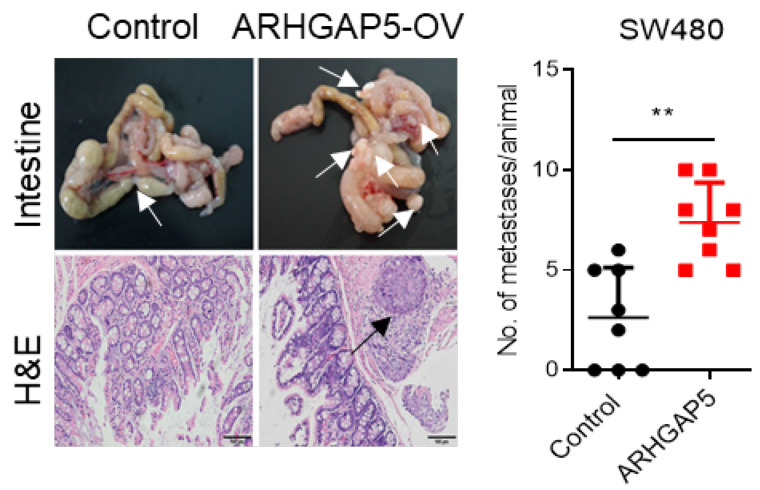
Correct image.

**Figure 6F:**
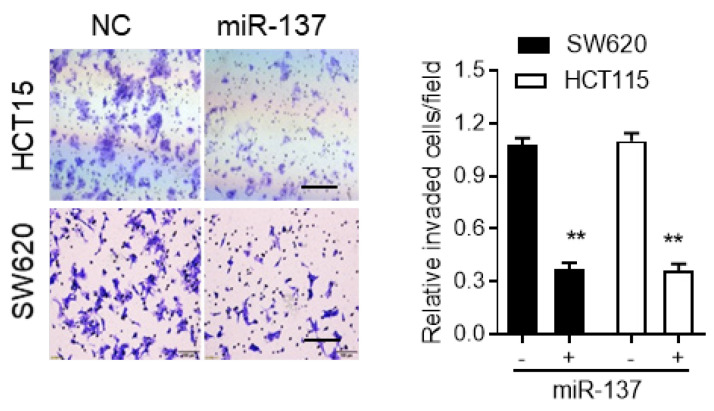
Correct image.

