# Supplementary material for: Investigation of the role and mechanism of ARHGAP5-mediated colorectal cancer metastasis
Source: Theranostics. 2020 May 1;10(13):5998–6010. doi: 10.7150/thno.43427 (PMC7254992; doi:10.7150/thno.43427)
Supplement: Supplementary file 1 — Supplementary figures and tables. [file thnov10p5998s1.pdf]

# Supplementary Methods

## RNA isolation and qPCR analysis

Total RNA was isolated with Trizol regent (Life Technologies, Carlsbad, USA) and then reversely transcribed to cDNA with a Takara kit (NHK, Japan) according to the manufacturer's instructions. The resulting complementary DNA was analyzed by qPCR performed with SYBR reagent using the IQ5 PCR system (Bio-Rad, Hercules, USA) in a LightCycler 480 instrument (Roche Diagnostics, Switzerland). β-Actin was used as the internal control gene and data were analyzed using the $2^{-\Delta\Delta ct}$ method. The sequences of used primers were described as following: ARHGAP5 (forward: 5'-agggaagctcaacgtagatgg-3', reverse: 5'-atgatccacgcattcatcacat-3.); CREB1 (forward: 5'-attcacaggagtcagtggatagt-3', reverse: 5'-caccgttacagtggtgatgg-3.); β-Actin (forward: 5'-catgtacgttgctatccaggc-3', reverse: 5'-ctccttaatgtcacgcacgat-3.).

## Immunofluorescence analysis

Immunoblotting and IHC analysis were conducted with standard procedures. Briefly, SW620 cells seeded in Glass Bottom Cell Culture Dish (NEST, Wuxi, China) were washed with PBS twice, fixed in 4% paraformaldehyde in PBS for 15 mins at room temperature and permeabilized with 0.25% Triton X-100. Samples were then incubated with 1% bovine serum albumin for 1 hour at room temperature followed by incubation with E-cadherin or N-cadherin antibody at 4 °C overnight. Samples were then incubated with secondary antibody for 1 h at room temperature. Finally, the sample was incubated with 4′,6-Diamidino-2-

phenylindole dihydrochloride (DAPI) for 3 mins. Images were obtained using a laser scanning confocal microscope FV1000 (Olympus, Tokyo, Japan) and analyzed using FluoViewer software.

**Lentiviral transduction and cell transfection**

Short hairpin RNA (shRNA) directed against ARHGAP5 (#1: gcagactgtgagagattatca; #2: gctgatagcaaagaggtatat) were ligated into the pLV12 vector (GenePharma, Shanghai, China). Lentiviruses were generated by transfecting lentiviral vector pLV12 together with packaging vector psPAX2 and envelope plasmid pMD2.G into 293T cells, and the infected cells were selected with puromycin for 7 days. An adenovirus expressing human wild type (WT) and mutant (Mu) ARHGAP5 were purchased from Hanbio Biotechnology Co. (Shanghai, China). The mutant ARHGAP5 (Mu) with a point mutation in conserved Arg residue as R1297A (Arg to Ala) [1, 2]. The SW480 or DLD-1 cells were infected with the adenovirus encoding ARHGAP5 and vector control according to the manufacturer's instructions. The small interfering RNAs (siRNAs) targeting CREB1 (#1: 5′-cuaacaaugacaaacgcua-3′; #2: 5′-gagcuguccucuccaguau-3′), RhoA (#1: 5′-ccuuauaguuacuguguaatt-3′; #2: 5′-aattatgtagcagatattgaa-3′), miR-137 mimic, miR-137-mut mimic and negative control (NC) oligonucleotides were obtained from Ribobio (Guangzhou, China). Cell transfections were performed using Lipofectamine 3000 (Invitrogen, Carlsbad, CA, USA) according to the manufacturer’s instructions.

**Chromatin immunoprecipitation (ChIP) assay**

ChIP assays were performed using a Chromatin Immunoprecipitation Assay Kit (Cat. #17-2, Millipore, Bedford, MA, USA) according to the manufacturer's

instructions as previously described [3]. CRC cells were grown to 80% confluence, and crosslinking was performed with 1% formaldehyde for 10 min. The cell lysates were sonicated to shear DNA to sizes of 300 to 1,000 bp. Equal aliquots of chromatin supernatants were incubated with anti-c-CREB1 or anti-IgG antibody overnight at 4°C with rotation. After reverse cross-link of protein/DNA complexes to free DNA, qRT-PCR was carried out to detect the CREB1 binding sites on ARHGAP5 promoter region. The sequences of used primers were described as following: Binding site 1 (BS1) (forward: 5'-aatggactcctcttctcgct-3', reverse: 5'- agctcgttcgcaggaacat-3); Binding site 2 (BS2) (forward: 5'-tggaggatgggactgaggag-3', reverse: 5'-ggagggagggggacgag-3).

**Luciferase assay**

DNA fragments from the 3'-UTR of ARHGAP5 that contained the predicted complementary sites of miR-137 were cloned into a pGL3-basic vector (Addgene, Cambridge, USA). Ten thousand CRC cells or 293T cells were seeded in triplicate in 48-well plates and allowed to settle for 24 h. Then, the pGL3-ARHGAP5-3'UTR reporter plasmids (100 ng) plus 5 ng of pRL-TK renilla plasmid (Promega, Madison, USA), which had increasing levels (10 and 50 nM) of negative control (NC), miR-137 mimic, or miR-137-mut mimic, were co-transfected into CRC cells using the Lipofectamine LTX reagent (Invitrogen, Carlsbad, USA) according to the manufacturer's instructions. Alternatively, luciferase pGL3-ARHGAP5 promoter (~2000 bp upstream of the start site) plasmid DNA (50 ng), and either vector control or fully sequenced c-CREB1 cDNA (50 ng) were co-transfected into 293T cells using the Lipofectamine LTX reagent. Luciferase and renilla signals were measured 24 h after transfection

using the Dual Luciferase Reporter Assay Kit (Promega, Madison, USA) according to the protocol provided by the manufacturer [4].

## References


1. N T, DA L, IG M. The function of the p190 Rho GTPase-activating protein is controlled by its N-terminal GTP binding domain. The Journal of biological chemistry. 1998; 273: 34631-8.
2. R L, B Z, Y Z. Structural determinants required for the interaction between Rho GTPase and the GTPase-activating domain of p190. The Journal of biological chemistry. 1997; 272: 32830-5.
3. Ju HQ, Ying H, Tian T, Ling J, Fu J, Lu Y, et al. Mutant Kras- and p16-regulated NOX4 activation overcomes metabolic checkpoints in development of pancreatic ductal adenocarcinoma. Nat Commun. 2017; 8: 14437.
4. Chen DL, Lu YX, Zhang JX, Wei XL, Wang F, Zeng ZL, et al. Long non-coding RNA UICLM promotes colorectal cancer liver metastasis by acting as a ceRNA for microRNA-215 to regulate ZEB2 expression. Theranostics. 2017; 7: 4836-49.

## Supplementary Tables

**Table S1**. Correlation between ARHGAP5 expression and clinicopathological features in 423 primary CRC.

| Parameters | Low expression N=240 | High expression N=183 | *P** |
|---|---|---|---|
| Age | | | |
| ＜57 | 125 (52.1%) | 96 (52.5%) | |
| ≥57 | 115 (47.9%) | 87 (47.5%) | 1.00 |
| Gender | | | |
| Male | 143 (59.6%) | 107 (58.9%) | |
| Female | 97 (40.4%) | 76 (41.1%) | 0.76 |
| Differentiation status | | | |
| Well/Moderate | 192 (80.0%) | 142 (77.6%) | |
| Poor and others | 48 (20.0%) | 41 (22.4%) | 0.55 |
| Tumor depth (T stage) | | | |
| m/sm/mp | 22 (9.2%) | 8 (4.4%) | |
| ss/se/si | 218 (90.8%) | 175 (95.6%) | 0.08 |
| Lymph node invasion (N stage) | | | |
| Absent | 86 (35.8%) | 81 (44.3%) | |
| Present | 154 (64.2%) | 102 (55.7%) | 0.08 |
| Vascular invasion | | | |
| Absent | 184 (76.7%) | 121 (66.4%) | |
| Present | 56 (23.3%) | 62 (33.9%) | 0.23 |
| Perineural invasion | | | |
| Absent | 119 (49.6%) | 94 (51.4%) | |
| Present | 121 (50.4%) | 89 (48.6%) | 0.76 |
| Clinical stage | | | |
| I, II | 75 (31.2%) | 74 (40.4%) | |
| III, IV | 165 (68.8%) | 109 (59.6%) | .0.05 |

**P* values determined by Chi-square test using SPSS 20.0. All statistical tests were two-sided. CRC=colorectal cancer; m=tumor invasion of mucosa; p=muscularis propria; sm=submocosa; ss=subserosa; se=serosa penetration; si=invasion to adjacent structures.

**Table S2.** Effect of factors on overall survival in CRC patients in the univariate and multivariate cox regression model.

| Factors | Univariate * | | Multivariate *, †, ‡ | |
|---|---|---|---|---|
| | HR (95% CI) | *P* | HR (95% CI) * | *P* |
| Age (<60/≥60) | 1.07 (0.73-1.58) | 0.72 | — | — |
| Gender (male/female) | 1.63 (1.07-2.46) | 0.02 | 1.36 (0.89-2.08) | 0.05 |
| Differentiation (poor/well, moderate) | 2.15 (1.43-3.24) | <0.001 | 1.64 (1.07-2.52) | 0.02 |
| Clinical Stage (III-IV/I-II) | 2.64 (1.64-4.27) | <0.001 | — | — |
| Tumor depth (m, sm, mp/ss, se, si) | 2.26 (0.83-6.16) | 0.10 | — | — |
| Lymph node invasion (present/absent) | 2.08 (1.35-3.22) | 0.001 | — | — |
| Vascular invasion (abnormal/normal) | 3.60 (2.44-5.30) | <0.001 | 1.84 (1.31-3.00) | 0.01 |
| Perineural invasion (abnormal/normal) | 2.95 (1.95-4.49) | <0.001 | — | — |
| Recrudesce (present/absent) | 5.62(3.80-8.31) | <0.001 | 3.89 (2.52-6.00) | <0.001 |
| ARHGAP5 (high/low) | 1.65 (1.12-2.44) | 0.01 | 1.78 (1.19-2.63) | 0.004 |

* Hazard ratios and *P* values were obtained from Cox proportional hazards regression. All statistical test were two-sided. † For the multivariate model, HR and *P* values were shown for the final set of stepwise selected variables only. ‡ The parameters with *P* value less than .05 in the univariate were included in the multivariate Cox analysis using SPSS 20.0. CRC=colorectal cancer; m= tumor invasion of mucosa; sm=submocosa; mp=muscularis propria; ss=subserosa; se=serosa penetration; si=invasion to adjacent structures.

# Supplementary Figure 1

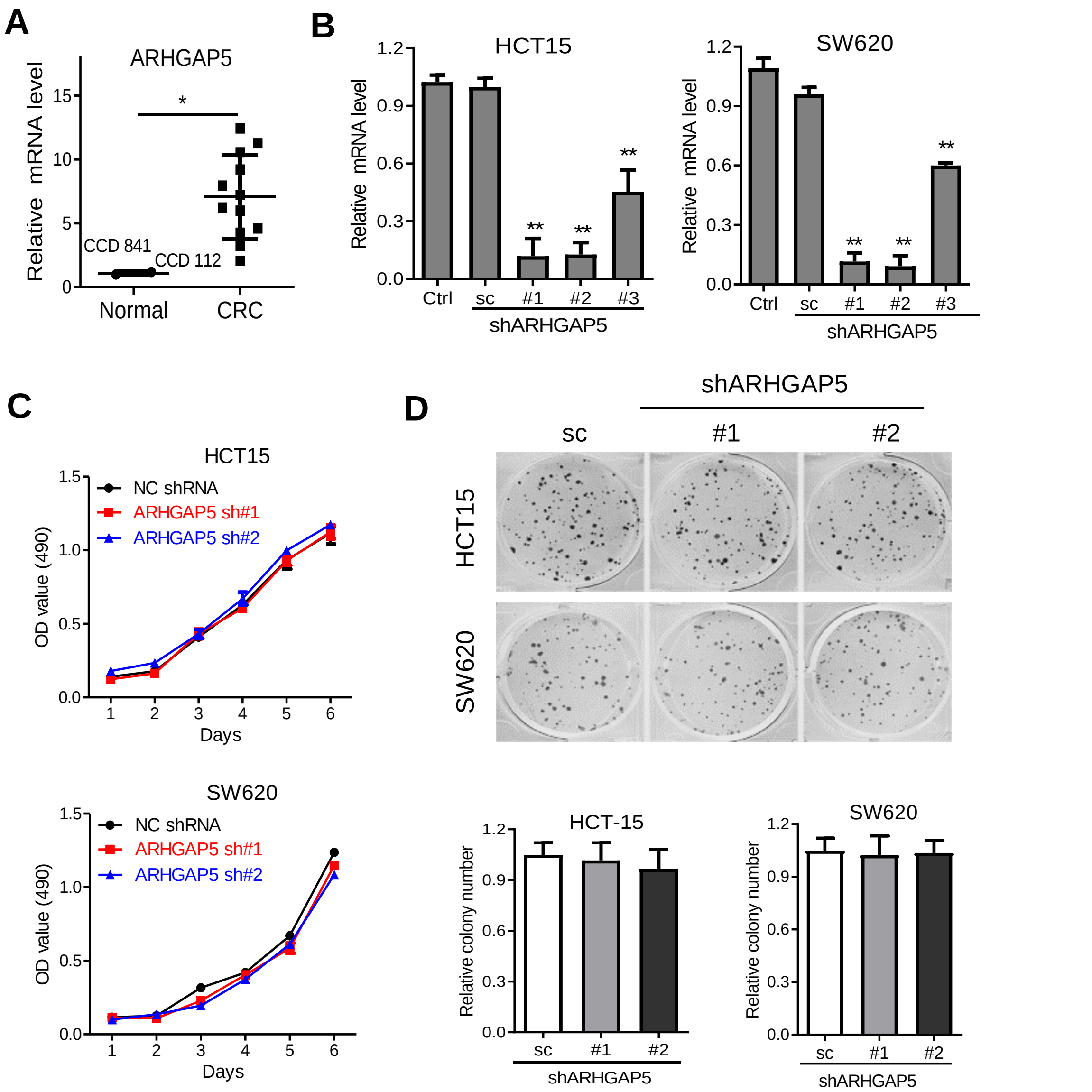


**Figure S1. Effects of ARHGAP5 inhibition on CRC cell growth and proliferation.** (A) qPCR analysis of ARHGAP5 expression in CRC and normal colon epithelial cell lines. (B) qPCR analysis evaluating the knockdown efficiency of ARHGAP5 with three unique shRNAs (#1, #2, and #3) in SW620 and HCT15 cells. (C) Growth curves of the indicated ARHGAP5-knockdown and control CRC cells, as measured by MTS assays. (D) Images and the quantification of colony formation by the indicated CRC cells with the knockdown of ARHGAP5. Data in A, B, C and D are presented as the mean ± the SD (n=3). ***P* < 0.01 (Student's *t*-test).

# Supplementary Figure 2

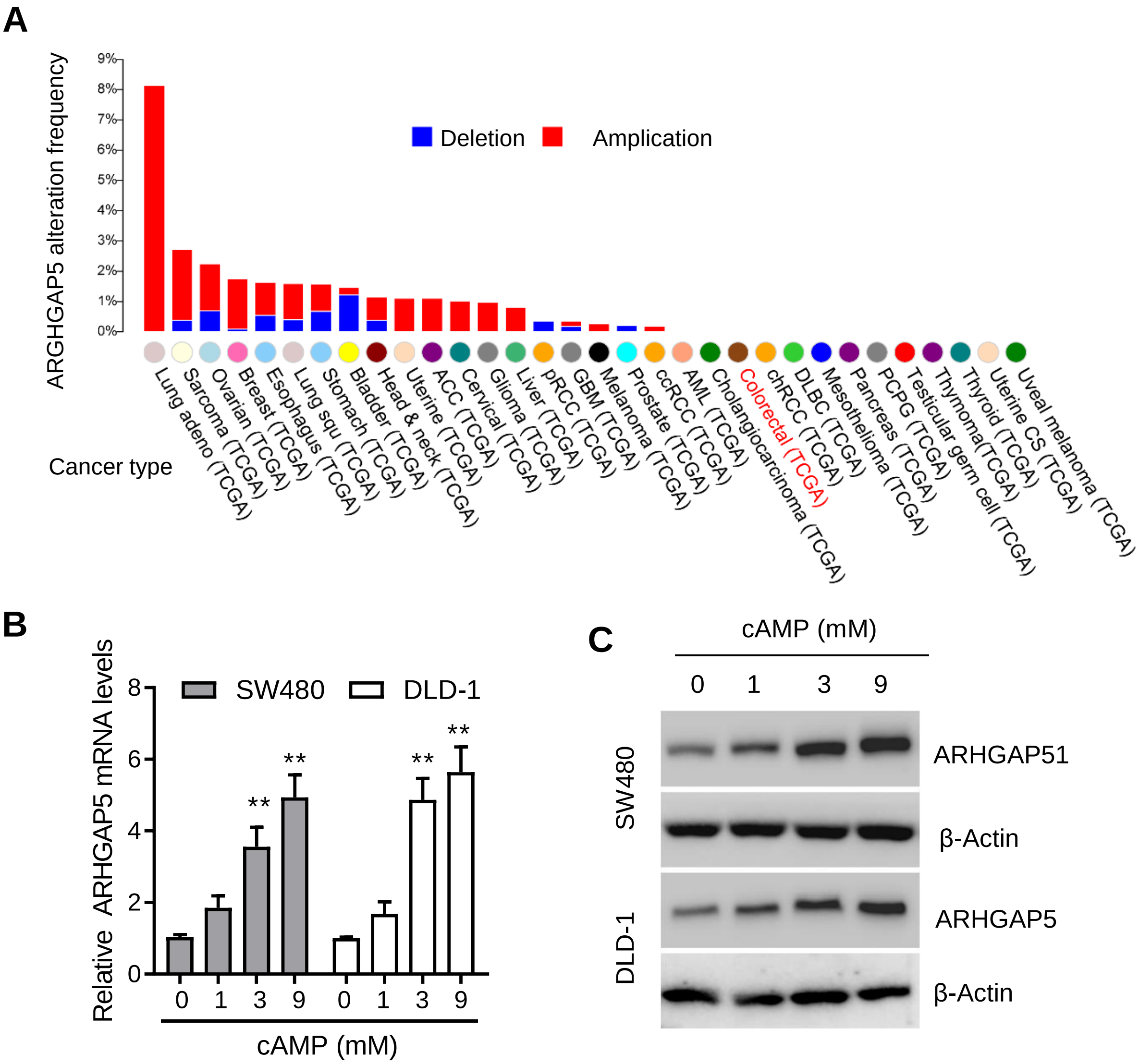


**Figure S2. ARHGAP5 is upregulated by cAMP.** (A) Distribution of the alteration frequency of ARHGAP5 in multiple cancer types. Details in parentheses indicate the source of the corresponding tumor dataset (cBioPortal for cancer genomics). (B-C) qPCR and immunoblotting analysis of ARHGAP5 expression in SW480 and DLD-1 cells cultured with medium alone or cAMP (1 mM, 3mM or 9mM, Sigma-Aldrich). β-Actin was included as a loading control. PCR data are presented as the mean ± the SD (n=3). ***P* < 0.01 (Student's *t*-test).

# Supplementary Figure 3

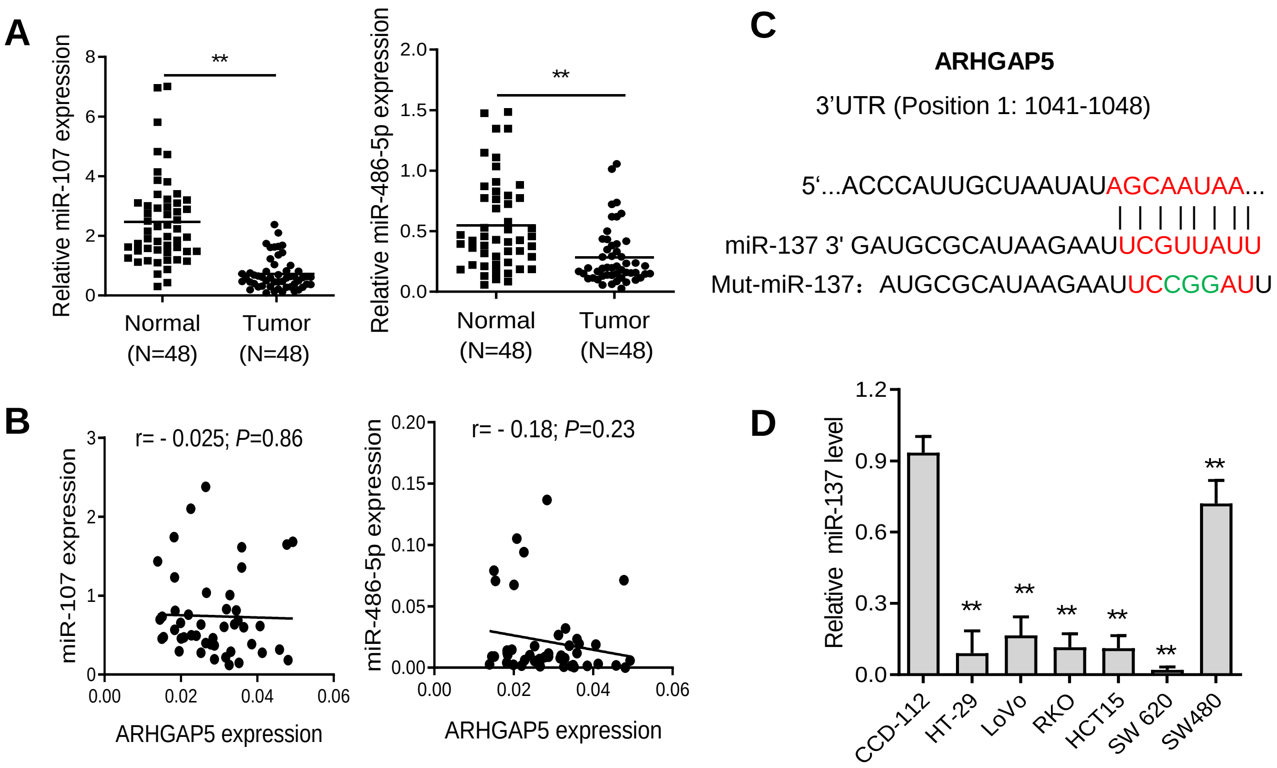


**Figure S3. Decreased miR-137 contributes to ARHGAP5 overexpression in CRC.** (A) qPCR analysis of miR-107 or miR-486-5p expression in paired CRC patient samples (n=48). (B) Scatterplots of ARHGAP5 vs miR-107 or miR-486-5p expression in CRC samples (n=48). The Pearson correlation coefficient (r) and *P* value are displayed. (C) Sequences of the indicated miR-137 and its potential binding site at the 3'UTR of ARHGAP5 are shown. (D) qPCR analysis of miR-137 expression in CRC and normal colon epithelial cell lines. ***P* < 0.01 versus control cells. Data are presented as the mean ± the SD (Student's *t*-test).
